# Veiled symmetry of disordered Parity-Time lattices: protected PT-threshold and the fate of localization

**DOI:** 10.1038/s41598-017-18589-z

**Published:** 2018-01-08

**Authors:** Andrew K. Harter, Franck Assogba Onanga, Yogesh N. Joglekar

**Affiliations:** Indiana University Purdue University Indianapolis (IUPUI), Indianapolis, 46202 USA

## Abstract

Open, non-equilibrium systems with balanced gain and loss, known as parity-time (${\mathscr{P}}{\mathscr{T}}$)-symmetric systems, exhibit properties that are absent in closed, isolated systems. A key property is the ${\mathscr{P}}{\mathscr{T}}$-symmetry breaking transition, which occurs when the gain-loss strength, a measure of the openness of the system, exceeds the intrinsic energy-scale of the system. We analyze the fate of this transition in disordered lattices with non-Hermitian gain and loss potentials ±*iγ* at reflection-symmetric sites. Contrary to the popular belief, we show that the ${\mathscr{P}}{\mathscr{T}}$-symmetric phase is protected in the presence of a periodic disorder which leads to a positive ${\mathscr{P}}{\mathscr{T}}$-symmetry breaking threshold. We uncover a veiled symmetry of such disordered systems that is instrumental for the said protection, and show that this symmetry leads to new localization behavior across the ${\mathscr{P}}{\mathscr{T}}$-symmetry breaking transition. We elucidate the interplay between such localization and the ${\mathscr{P}}{\mathscr{T}}$-symmetry breaking phenomena in disordered ${\mathscr{P}}{\mathscr{T}}$-symmetric lattices, with Hermitian disorder or gain-loss disorder, and support our conclusions with a beampropagation- method analysis. Our theoretical predictions provide avenues for experimental realizations of -symmetric systems with engineered disorder.

## Introduction

Over the past decade, classical and quantum open systems in two categories have been intensely investigated for their non-equilibrium properties. The first category consists of systems that are in quasi-equilibrium and can be studied using linear response theory^1^. The second category has systems that are far removed from equilibrium^2^, making perturbative methods inapplicable. Open systems with balanced gain and loss, called parity-time (${\mathscr{P}}{\mathscr{T}}$)-symmetric systems, straddle the two categories. In the quantum context, ${\mathscr{P}}{\mathscr{T}}$-symmetric systems refer to those described by a non-Hermitian Hamiltonian ${H}_{PT}\ne {H}_{PT}^{\dagger }$ that is invariant under combined parity (${\mathscr{P}}$) and time-reversal (${\mathscr{T}}$) operations and leads to a non-unitary time evolution. The non-degenerate spectrum of *H*_*PT*_ is purely real when the non-Hermiticity is small and becomes complex-conjugate pairs when it exceeds a threshold set by the Hermitian part of the Hamiltonian. This transition is called the ${\mathscr{P}}{\mathscr{T}}$-symmetry breaking transition^3^. In the ${\mathscr{P}}{\mathscr{T}}$-symmetric phase (real spectrum), the system is in a quasi-equilibrium state characterized by bounded, periodic oscillations in the system particle number. In the ${\mathscr{P}}{\mathscr{T}}$-broken phase (complex spectrum), the system is far removed from equilibrium, and the particle number increases exponentially with time^4^.

Two decades ago, ${\mathscr{P}}{\mathscr{T}}$-symmetric Hamiltonians were first studied for continuum models on an infinite line^5–7^. The past five years, however, have made it clear that the experimentally relevant ones^8–12^ are discrete lattice models^13–16^ or continuum models on a finite line^17–19^. For a one dimensional lattice with *N* sites, the parity operator represents reflection about the lattice center, i.e., ${{\mathscr{P}}}_{mn}={\delta }_{m\bar{n}}$ where $\bar{n}=N+1-m$ is the reflection-counterpart of site *n*. The time-reversal operator is given by complex conjugation, ${\mathscr{T}}=\ast$. A typical ${\mathscr{P}}{\mathscr{T}}$-symmetric Hamiltonian consists of a Hermitian part *H*_0_ that represents kinetic energy and a non-Hermitian part Γ that represents balanced gain and loss. The ${\mathscr{P}}{\mathscr{T}}$-symmetric nature of *H*_0_ itself implies that its eigenfunctions are either symmetric or antisymmetric, ensures that the odd-order perturbative corrections from the gain-loss potential Γ to the eigenenergies of *H*_0_ vanish^20^, and thus leads to a positive ${\mathscr{P}}{\mathscr{T}}$-symmetry breaking threshold. Recall that in two dimensions or higher, the spectrum of *H*_0_ is degenerate and therefore, generically, the ${\mathscr{P}}{\mathscr{T}}$-breaking threshold is zero^21,22^ unless the gain-loss potential has no matrix elements between within a degenerate subspace^23^.

Discrete ${\mathscr{P}}{\mathscr{T}}$-symmetric lattice Hamiltonians have been realized in coupled resonators^10–12^ and coupled optical waveguides with balanced gain and loss^9^. Evanescently coupled optical waveguides are also an exceptional platform for simulating key quantum phenomena^24^ including Bloch oscillations^25^ and Anderson localization in one dimension due to arbitrarily weak disorder^26^. Although initially predicted in the condensed-matter context^27–30^, these phenomena have been thoroughly investigated in waveguide lattices because the Maxwell wave equation, under paraxial approximation, is isomorphic to the Schrödinger equation for the wave-envelope function |*ψ*(*t*)〉^24^. In a sharp contrast with the nature-given lattices in condensed matter systems, waveguide lattices can be fabricated with a wide range of site-to-site tunneling amplitudes and on-site potentials; local or long-ranged “impurity” potentials; and on-site or tunneling disorder. This versatility has permitted the observation of disorder-induced localization, its insensitivity to the source of the disorder, as well as the signatures of the disorder-source in Hanbury-Brown Twiss correlations in disordered waveguide lattices^31^ and fibers^32^.

What is the fate of a disordered ${\mathscr{P}}{\mathscr{T}}$-symmetric system? In general, the ${\mathscr{P}}{\mathscr{T}}$-symmetric phase is fragile in the sense that an arbitrarily weak disorder - Hermitian or otherwise - suppresses the symmetry-breaking threshold to zero^13,20^. It does so because a random disorder does not preserve the symmetries of the underlying Hamiltonian. A straightforward way to salvage the fragile ${\mathscr{P}}{\mathscr{T}}$-symmetric phase is to require a ${\mathscr{P}}{\mathscr{T}}$-symmetric disorder^33^. However, this approach imposes highly non-local correlations on the randomness and is therefore difficult to implement, even with an engineered disorder. Thus questions about localization and ${\mathscr{P}}{\mathscr{T}}$-symmetry breaking in a disordered ${\mathscr{P}}{\mathscr{T}}$-symmetric system appear moot^34,35^.

In this report, we show that the ${\mathscr{P}}{\mathscr{T}}$-symmetric phase in a disordered system is not always fragile, and that it is protected against random tunneling or on-site potential disorder if the disorder has specific periodicities. We elucidate an underlying symmetry that is critical for the said protection. We investigate the distribution of ${\mathscr{P}}{\mathscr{T}}$-breaking threshold in such disordered systems and its dependence on the nature (tunneling or on-site potential) and the distribution (Gaussian, uniform, etc.) of disorder. In Hermitian disordered systems, disorder-averaged single particle properties, such as density of states and the localization profile, do not depend upon these details. Here, we show that the distribution of ${\mathscr{P}}{\mathscr{T}}$-symmetry breaking threshold is sensitive to those Hermitian-disorder attributes, whereas for a gain-loss disorder, it is not. Our results demonstrate that a disordered ${\mathscr{P}}{\mathscr{T}}$-symmetric system exhibits novel properties absent in its Hermitian counterpart.

## Disordered lattice model

Consider an *N*-site tight-binding lattice with gain and loss potentials ±*iγ* located at parity symmetric sites *m*_0_ ≤ *N*/2 and ${\bar{m}}_{0} > N/2$ respectively; the lattice has open boundary conditions, meaning the first and the *N*th site has only one neighbor each. The distance between the gain and the loss sites, $d={\bar{m}}_{0}-{m}_{0}$, ranges from *N* − 1 to one (two) when *N* is even (odd). The non-Hermitian, ${\mathscr{P}}{\mathscr{T}}$-symmetric Hamiltonian for this lattice is given by *H*_*PT*_ = *H*_0_ + Γ where

1 $${H}_{0}=-J\,\sum _{k=1}^{N-1}\,(|k\rangle \langle k+\mathrm{1|}+|k+1\rangle \langle k|)={H}_{0}^{\dagger },$$

2 $${\rm{\Gamma }}=i\gamma (|{m}_{0}\rangle \langle {m}_{0}|-|{\bar{m}}_{0}\rangle \langle {\bar{m}}_{0}|)=-{{\rm{\Gamma }}}^{\dagger }\mathrm{.}$$

*J* > 0 is the constant tunneling amplitude that sets the energy-scale for the Hermitian Hamiltonian *H*_0_ and |*k*〉 is a single-particle state localized at lattice site *k*. Since the Hamiltonian *H*_*PT*_ commutes with the antilinear operator ${\mathscr{P}}{\mathscr{T}}$, it follows that its spectrum is either purely real or consists of complex conjugate pairs^36,37^. The spectrum is real when *γ* ≤ *γ*_*PT*_(*m*_0_) where the *γ*_*PT*_(*m*_0_) denotes the gain-location dependent ${\mathscr{P}}{\mathscr{T}}$-symmetry breaking threshold. When *N* is even, the threshold is maximum when the gain and loss potentials are nearest to each other or farthest away from each other, i.e., *γ*_*PT*_ = *J* when *d* = 1 and *d* = *N* − 1. When *N* is odd, *γ*_*PT*_ → *J*/2 when *d* = 2 and *γ*_*PT*_ → *J* when *d* = *N* − 1. This unexpected robustness of the ${\mathscr{P}}{\mathscr{T}}$-symmetry breaking threshold at the largest gain-loss distance is due to open boundary conditions^38,39^. In the presence of a random, uncorrelated disorder, the threshold is suppressed to zero. In the following subsection, we show that introducing a periodic disorder alleviates this problem.

### ${\bf{P}}{\bf{T}}$ phase diagram of a disordered lattice

We consider two classes of Hermitian disorders, one in the tunneling amplitude and the second in the on-site potential, each with lattice period *p*,

3 $${V}_{T}(\lambda )=J\lambda \,\sum _{k=1}^{N-1}\,{r}_{k}(|k\rangle \langle k+\mathrm{1|}+|k+1\rangle \langle k|),$$

4 $${V}_{O}({\rm{\Delta }})=J{\rm{\Delta }}\,\sum _{k=1}^{N}\,{r}_{k}|k\rangle \langle k\mathrm{|.}$$

**Figure 1:**
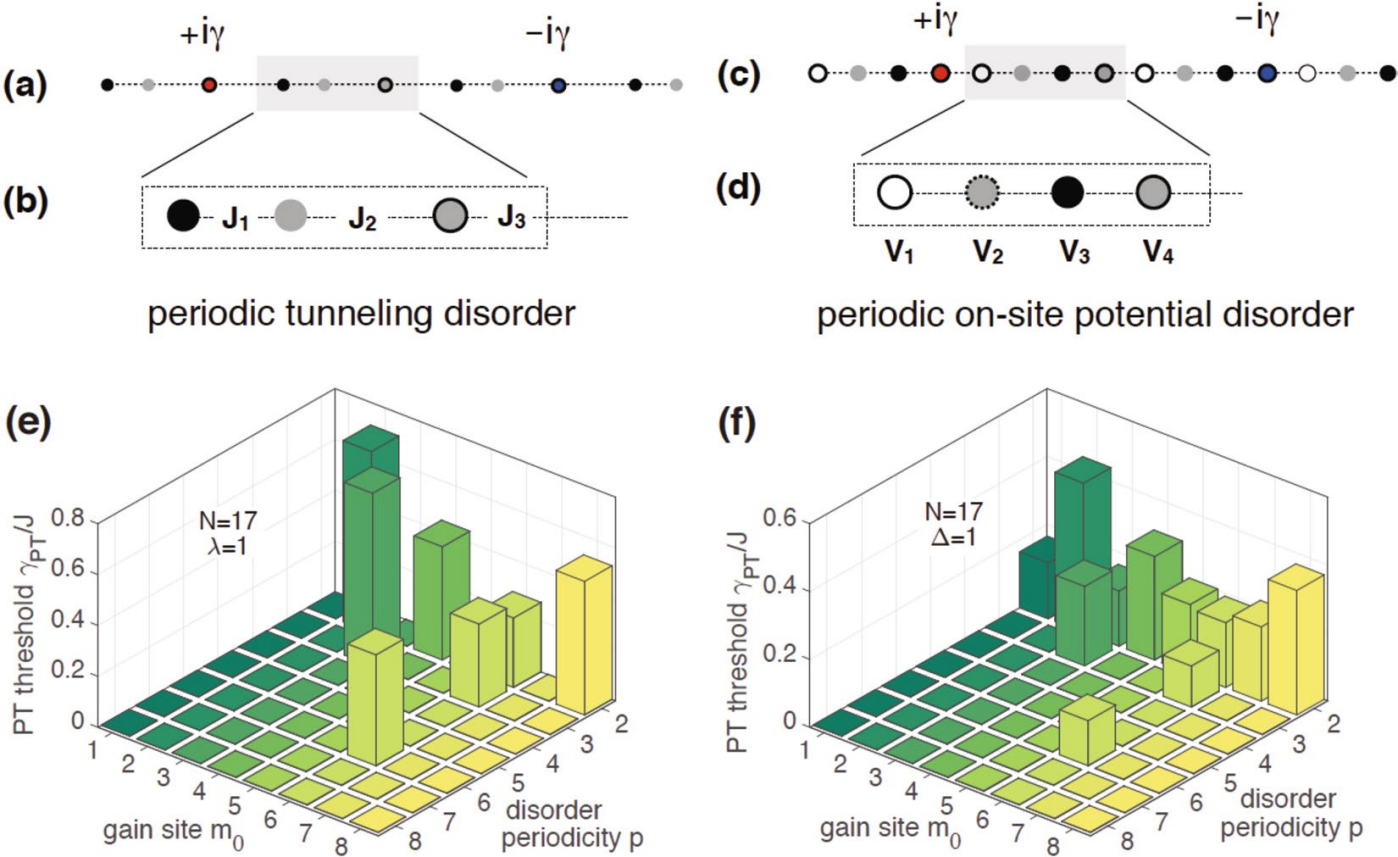
Disordered ${\mathscr{P}}{\mathscr{T}}$-symmetric lattices with open boundary conditions. (**a**) An 11-site lattice with gain potential +*iγ* at site *m*_0_ = 3 and random periodic tunneling *J*_*k*_ = *J*(1 + *λr*_*k*_). Here *λ* = 1 is the strength of the disorder and {*r*_1_, …, *r*_*p*_} are *p* random numbers with zero mean and unit variance. (**b**) The tunneling disorder has period *p* = 3. (**c**) A 15-site lattice with uniform tunneling, *m*_0_ = 4, and random on-site potentials *V*_*k*_ = *J*Δ*r*_*k*_ with Δ = 1. (**d**) The potential disorder has period *p* = 4. (**e**) ${\mathscr{P}}{\mathscr{T}}$-symmetry breaking threshold *γ*_*PT*_(*m*_0_) as a function of gain site *m*_0_ ≤ *N*/2 and tunneling disorder period *p* ≤ *N*/2 for an *N* = 17 site lattice shows that *γ*_*PT*_ > 0 when *N* + 1 = 0 mod *p* and *m*_0_ = 0 mod *p*; it is zero otherwise. (**f**) Results for on-site disorder show the same behavior except at *p* = 2 for an odd *N*. Then the on-site disorder is ${\mathscr{P}}{\mathscr{T}}$ symmetric, and *γ*_*PT*_(*m*_0_) > 0 for all *m*_0_. These results imply that the positive ${\mathscr{P}}{\mathscr{T}}$-breaking threshold of a uniform lattice is protected from a periodic disorder under the right circumstances.

The dimensionless numbers *λ* ≥ 0 and Δ ≥ 0 represent the strength of tunneling and on-site disorder respectively, {*r*_1_, …, *r*_*p*_} are independent, identically distributed (i.i.d.) random numbers with zero mean and unit variance, and the periodic nature of disorder implies that *r*_*k*′_ = *r*_*k*_ if *k*′ − *k* = 0 mod *p*. We remind the reader that although the randomness of disorder is only confined to a unit-cell of size *p*, the lattice with *N* sites may or may not contain integer number of such unit cells. The existence of a finite threshold depends critically on these details and, thus, cannot be obtained via the Bloch-theorem approach. Figure 1(a,b) show the schematic of a disordered lattice with *N* = 11 sites and gain potential *iγ* at site *m*_0_ = 3. The tunneling disorder *V*_*T*_ has period *p* = 3, and the three independent, random tunnelings within a unit cell are given by *J*_*k*_ = *J*(1 + *λr*_*k*_). Figure 1(c,d) show an on-site-potential disordered lattice with *N* = 15 sites, gain potential at site *m*_0_ = 4, and disorder period *p* = 4; the four independent, random potentials within a unit cell are given by *V*_*k*_ = *J*Δ*r*_*k*_. Note that the periodic disorder potential in each case is not ${\mathscr{P}}{\mathscr{T}}$-symmetric. Therefore, conventional wisdom suggests that the ${\mathscr{P}}{\mathscr{T}}$-symmetry breaking threshold for the disordered Hamiltonian in each case will be zero.

Figure 1(e) shows the numerically obtained threshold *γ*_*PT*_(*m*_0_, *p*) for an *N* = 17 lattice with tunneling disorder strength *λ* = 1. Its key features are as follows. The threshold *γ*_*PT*_ is nonzero only when *N* + 1 and *m*_0_ are multiples of disorder period *p*. Thus, when *p* = 2 the threshold is nonzero only when *m*_0_ is even, for *p* = 3 it is nonzero for *m*_0_ = {3, 6}, and for *p* = 6, it is nonzero only when *m*_0_ = 6. It is identically zero for periods *p* = {4, 5, 7, 8} for any gain-site location *m*_0_. These results, obtained for a particular realization of the tunneling disorder, are generic. They show that a tunneling disorder with appropriate period *p* and gain locations *m*_0_ leads to a positive ${\mathscr{P}}{\mathscr{T}}$-symmetry breaking threshold with values comparable to that of a clean system, ${\gamma }_{PT}\sim J$.

Figure 1(f) shows the corresponding results for an on-site disorder with strength Δ = 1. The salient features of the phase diagram are the same: *γ*_*PT*_ > 0 when *N* + 1 = 0 mod *p* and *m*_0_ = 0 mod *p*. Thus, periodicities *p* = {2, 3, 6} have a positive threshold for appropriate gain locations, while *γ*_*PT*_ = 0 for all other disorder periods. In addition, when *p* = 2 (on-site, dimer disorder), the symmetry breaking threshold is nonzero for odd values of gain location as well. This is the only qualitative difference between the threshold results for tunneling vs. on-site disorders. It arises because for an odd *N* and *p* = 2, the on-site disorder is always ${\mathscr{P}}{\mathscr{T}}$-symmetric, i.e., $[{\mathscr{P}}{\mathscr{T}},{V}_{O}]=0$. For an even lattice, both tunneling and on-site dimer disorders have *γ*_*PT*_ > 0 only when the gain potential site is even.

Results in Fig. 1(e,f) are surprising because *they show that the symmetry breaking threshold is robust against disorders that are not reflection symmetric* ^40^. They hint at the existence of another antilinear operator that commutes with the disordered Hamiltonian^36,37^. In the next subsection, we uncover this symmetry and discuss its signatures.

### The Π-operator and a veiled symmetry

The tunneling Hamiltonian of a uniform lattice can be expressed as *H*_0_ = *UDU* ^†^ where *D*_*αβ*_ = *ε*_*α*_ *δ*_*αβ*_ = −2*J* cos *p*_*α*_ *δ*_*αβ*_ is the eigenvalues matrix, the unitary matrix has entries ${U}_{m\alpha }=\sqrt{2/(N+\mathrm{1)}}\,\sin ({p}_{\alpha }m)$, and *p*_*α*_ = *πα*/(*N* + 1) are the quasimomenta consistent with open boundary conditions. The spectrum of *H*_0_ is particle-hole symmetric, ${\varepsilon }_{\bar{\alpha }}=-{\varepsilon }_{\alpha }$, and its eigenfunctions satisfy ${U}_{\bar{m}\alpha }={(-\mathrm{1)}}^{\alpha -1}{U}_{m\alpha }$ and ${U}_{m\bar{\alpha }}={(-\mathrm{1)}}^{m-1}{U}_{m\alpha }$. Here $\bar{\alpha }=N+1-\alpha$ is the particle-hole counterpart of the eigenvalue index *α*. For a given *H*_0_, one can generate a family of operators *P* = *USU* ^†^ where *S* = diag(±, …, ±1) is a diagonal matrix with randomly chosen entries ±1; there are 2^*N*−1^ such distinct operators. When *S* = 1_*N*_ the result is the identity and when ${S}_{kk^{\prime} }={(-\mathrm{1)}}^{k-1}{\delta }_{kk^{\prime} }={\mathscr{S}}$, the result is the reflection operator ${\mathscr{P}}$. This procedure generalizes to the case of a disordered Hermitian Hamiltonian

5 $${H}_{0}(\lambda ,{\rm{\Delta }})={H}_{0}+{V}_{T}\,(\lambda )+{V}_{O}({\rm{\Delta }})$$

and leads to 2^*N*−1^ *disorder*-*dependent operators P*(*λ*, Δ). It is easy to show that *P* = *P* ^†^ = *P* ^−1^ and *P*(*λ*, Δ) commutes with *H*_0_(*λ*, Δ). However, in general, the operator ${\rm{\Pi }}(\lambda ,{\rm{\Delta }})=U(\lambda ,{\rm{\Delta }}){\mathscr{S}}{U}^{\dagger }(\lambda ,{\rm{\Delta }})$ does not equal the reflection operator on the lattice.

**Figure 2:**
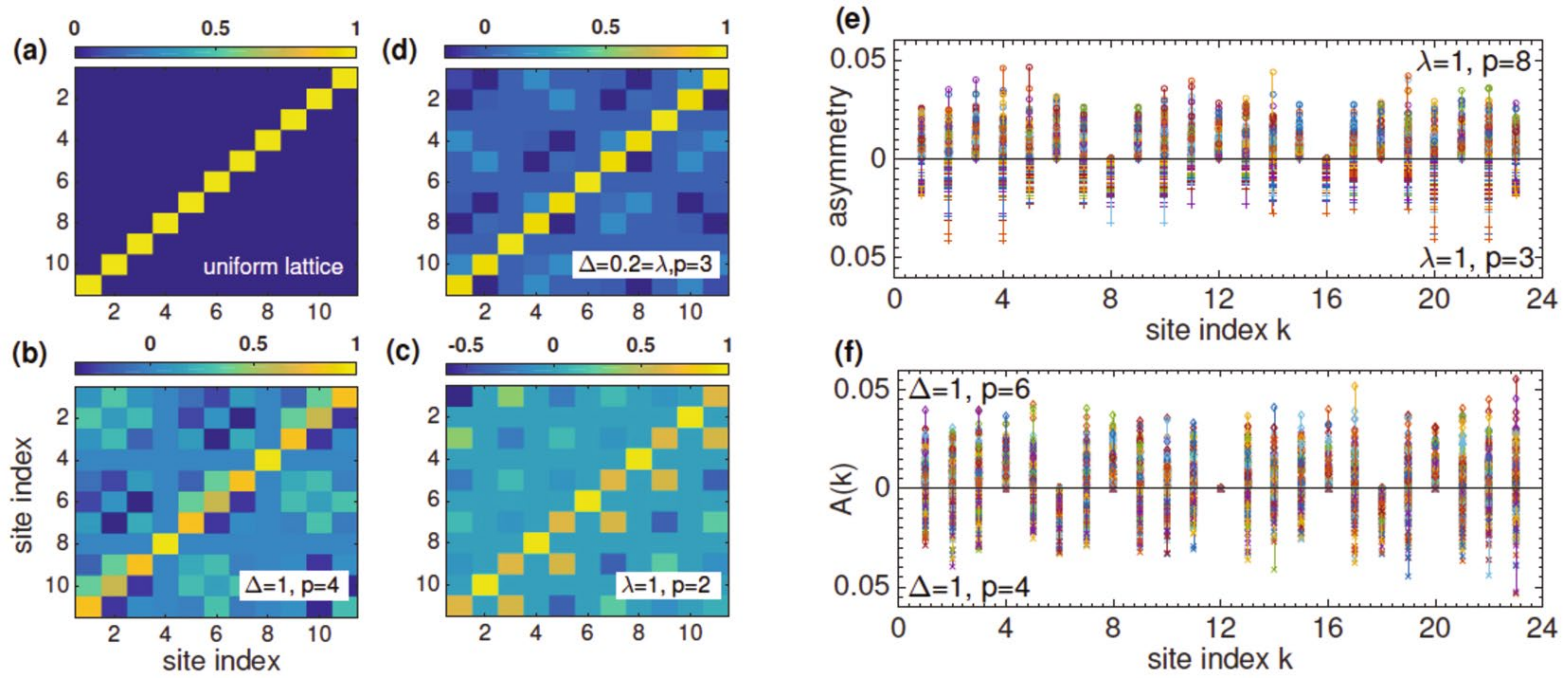
Veiled symmetry of a disordered lattice. (**a**) For a uniform lattice, the Π-operator is the same as a reflection. Typical parity operators Π for an *N* = 11 site lattice with different disorder strengths *λ*, Δ and periods *p* are shown in (**b–d**). In each case ${{\rm{\Pi }}}_{k\bar{k}}=1$ if and only if *N* + 1 and *k* are multiples of *p*, and ∑_*k*′_ Π_*kk*′_ = 1 otherwise. (**e**) Disorder- and site-dependent asymmetry functions *A*(*k*) for an *N* = 23 site lattice with *M* = 100 disorder realizations. The tunneling disorder strength is *λ* = 1. The asymmetry vanishes only if the disorder period *p* satisfies *N* + 1 = 0 mod *p* and only on sites *k* that are multiples *p*. (**f**) Results for an on-site disorder with strength Δ = 1 show the same quantitative trend. Note that the vertical axis in the bottom panels for (**e**,**f**) is inverted. This veiled symmetry of the eigenfunctions of *H*_0_(*λ*, Δ) is instrumental to the positive ${\mathscr{P}}{\mathscr{T}}$-symmetry breaking thresholds in Fig. 1.

Figure 2(a–d) show typical features of the Π operator in the site basis. For a clean system, ${\rm{\Pi }}={\mathscr{P}}$, panel (a). In the presence of disorder, Π is not a sparse matrix. Note that it satisfies ${{\rm{\Pi }}}_{kk^{\prime} }={\delta }_{k\bar{k}}={{\mathscr{P}}}_{k\bar{k}}$ if and only if the site labels *k*, *k*′ are both multiples of the disorder period *p*. When *k* ≠ 0 mod *p*, the unit weight is distributed to other elements in the same column. These results are generic and apply for on-site potential disorder, panel (b); tunneling disorder, panel (c); or a combination of the two, panel (d). In all cases, the ${\rm{\Pi }}{\mathscr{T}}$ operator commutes with the Hamiltonian *H*_0_(*λ*, Δ). A positive symmetry-breaking threshold, then, is possible if and only if the antilinear operator ${\rm{\Pi }}{\mathscr{T}}$ also commutes with the gain-loss potential Γ, eq. (2). It is straightforward, albeit tedious, to verify that it is so only when *N* + 1 and *m*_0_ are integer multiples of the disorder period.

What does the Π operator represent? An insight into its structure is offered by the simplest example where an explicit, analytical expression for the Π operator can be obtained. Let us consider an *N* = 5 site lattice and tunneling disorder with period *p* = 2. Without loss of generality, this system is mapped onto a dimer model with alternating tunneling amplitudes given by *J*_*r*_(1 ± *λ*) where *J*_*r*_ is the average tunneling amplitude for a dimer and *λ* is proportional to the variance of the disorder. It is straightforward to obtain the Π operator in this case,

6 $${\rm{\Pi }}(\lambda )=\tfrac{1}{1+3{\lambda }^{2}}\,[\begin{array}{ccccc}-2\lambda \mathrm{(1}-\lambda ) & 0 & 2\lambda \mathrm{(1}+\lambda ) & 0 & \mathrm{(1}-{\lambda }^{2})\\ 0 & 0 & 0 & \mathrm{(1}+3{\lambda }^{2}) & 0\\ 2\lambda \mathrm{(1}+\lambda ) & 0 & \mathrm{(1}-{\lambda }^{2}) & 0 & -2\lambda \mathrm{(1}-\lambda )\\ 0 & \mathrm{(1}+3{\lambda }^{2}) & 0 & 0 & 0\\ \mathrm{(1}-{\lambda }^{2}) & 0 & -2\lambda \mathrm{(1}-\lambda ) & 0 & 2\lambda \mathrm{(1}+\lambda )\end{array}]\mathrm{.}$$

We see that Eq. (6) reduces to the reflection operator when *λ* = 0. For *λ* > 0 and an odd *k*, the unit weight at ${{\rm{\Pi }}}_{k\bar{k}}$ is distributed to other elements in the same row, ${\sum }_{k^{\prime} }\,{{\rm{\Pi }}}_{kk^{\prime} }(\lambda )=1$. These properties are consistent with those shown in Fig. 2(b–d).

An complementary insight into the vanishing commutator, $[{\rm{\Gamma }}({m}_{0}),{\rm{\Pi }}{\mathscr{T}}]=0$, is offered by the effect of periodic disorder on the eigenfunctions of the uniform lattice. When the disorder is zero, the eigenfunctions *U*_*mα*_ are symmetric or antisymmetric, i.e., ${U}_{\bar{m}\alpha }={(-\mathrm{1)}}^{\alpha -1}{U}_{m\alpha }$. This property ensures that odd-order perturbative corrections due to the gain-loss potential ±*iγ* vanish, and leads to a positive ${\mathscr{P}}{\mathscr{T}}$ breaking threshold^20^. Are the eigenfunctions of the disordered Hamiltonian *H*_0_(*λ*, Δ) also reflection symmetric? To address this question, we define a disorder- and site-dependent asymmetry function

7 $$A(k;\lambda ,{\rm{\Delta }})=\sum _{\alpha =1}^{N}\,|{U}_{\bar{k}\alpha }(\lambda ,{\rm{\Delta }})+{(-\mathrm{1)}}^{\alpha }{U}_{k\alpha }(\lambda ,{\rm{\Delta }})|.$$

It follows that *A* ≥ 0 in general and for a uniform lattice, *A*(*k*) ≡ 0. The asymmetry functions *A*(*k*) for *M* = 100 different disorder realizations on an *N* = 23 site lattice are shown in Fig. 2(e,f). Note that the vertical axis in the bottom panel for both figures is inverted. When the tunneling disorder period is *p* = 8, *A*(*k*) = 0 only at sites *k* = {8, 16} (top panel), whereas when *p* = 3 the function vanishes exactly when *k* = 0 mod 3 (bottom panel). Figure 2(f) has the corresponding results for an on-site disorder with period *p* = 6 (top panel) and *p* = 4 (bottom panel). Once again, we see that *A*(*k*) = 0 if and only if the site index is a multiple of *p*. The asymmetry function is nonzero everywhere when the disorder period and lattice size do not satisfy *N* + 1 = 0 mod *p*. Results in Fig. 2(e,f) show that the disordered eigenfunctions *U*_*mα*_(*λ*, Δ) are neither symmetric nor antisymmetric, but, when restricted to specific sites, they show these symmetries^40^. Thus, although the Hamiltonian *H*_*PT*_(*λ*, Δ) = *H*_0_(*λ*, Δ) + Γ is not ${\mathscr{P}}{\mathscr{T}}$-symmetric, it is ${\rm{\Pi }}{\mathscr{T}}$-symmetric under these constraints. This veiled symmetry of the eigenfunctions of disordered Hamiltonian *H*_0_(*λ*, Δ) gives rise to the positive ${\mathscr{P}}{\mathscr{T}}$ breaking thresholds seen in Fig. 1.

### Disorder induced ${\mathscr{P}}{\mathscr{T}}$ threshold distribution and localization

Disordered models with positive ${\mathscr{P}}{\mathscr{T}}$-symmetry breaking thresholds prompt a number of questions. How does the probability distribution function of the ${\mathscr{P}}{\mathscr{T}}$-breaking threshold *PDF*(*γ*_*PT*_) depend on the strength of the disorder? Does it depend on the distribution of the disorder? Is it different for on-site and tunneling disorders? What is the fate of localization in ${\mathscr{P}}{\mathscr{T}}$-symmetric systems? These questions are addressed in the following paragraphs.

**Figure 3:**
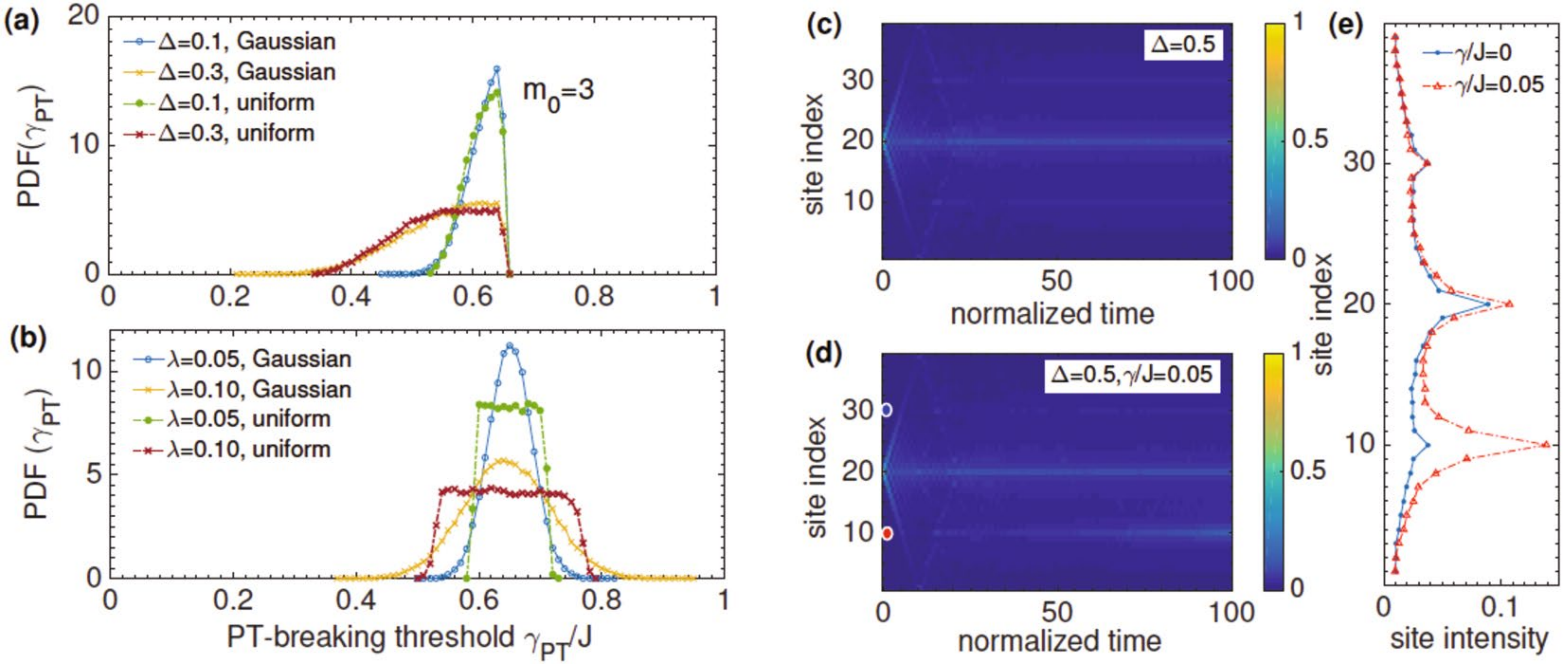
${\mathscr{P}}{\mathscr{T}}$-symmetry breaking threshold distribution *PDF*(*γ*_*PT*_) for the gain potential at site *m*_0_ = 3, in an *N* = 17 site lattice with disorder period *p* = 3. (**a**) For an on-site potential disorder, the threshold distribution *PDF*(*γ*_*PT*_) broadens as disorder strength Δ increases and it is independent of the disorder distribution, Gaussian or uniform. (**b**) For the tunneling disorder, the threshold distribution *PDF*(*γ*_*PT*_) mimics the disorder distribution, giving different results for a Gaussian disorder and the uniform disorder. (**c**) Localization in an *N* = 39 site lattice, with on-site disorder period *p* = 10, the initial state at the center of the lattice, and *M* = 10^3^ disorder realizations. When *γ* = 0, the disorder-averaged intensity *I*_*d*_(*k*, *t*) shows satellite peaks at *k* = *k*_0_ mod *p* in addition to the usual peak at the initial site *k*_0_ = 20. (**d**) when the gain-potential is turned on, *γ*/*J* = 0.05, intensity weight at the gain site *m*_0_ = 10 increases with time. (**e**) Intensity profile *I*_*d*_(*k*, *t*) at time *Jt* = 100 shows that the increase in the intensity at the gain-site when *γ* > 0 (red open triangles) does not come at the expense of the intensity at other sites, but instead from the non-unitary time evolution.

Figure 3 shows *PDF*(*γ*_*PT*_) in the presence of on-site potential disorder, panel (a), and tunneling disorder, panel (b). The results are for the ${\mathscr{P}}{\mathscr{T}}$-symmetry breaking threshold at gain site *m*_0_ = 3 in an *N* = 17 lattice, obtained by using *M* = 5 × 10^4^ realizations of disorder with period *p* = 3. We remind the reader that when *N* + 1 is not a multiple of the disorder period *p*, the ${\mathscr{P}}{\mathscr{T}}$ breaking threshold is zero for all gain locations, and even when the constraint is satisfied, *γ*_*PT*_(*m*_0_) = 0 for all locations that are not multiples of the period *p*. Thus, in the following, we only focus on configurations that lead to a positive ${\mathscr{P}}{\mathscr{T}}$ symmetry breaking threshold. The horizontal axis in each panel is the dimensionless threshold *γ*_*PT*_/*J*. Panel (a) shows that as the on-site disorder strength Δ increases, the threshold distribution *PDF*(*γ*_*PT*_) becomes broader, and skewed towards values smaller than its clean-limit value. In addition, *PDF*(*γ*_*PT*_) is independent of the disorder distribution, i.e., it is the same whether the random, periodic potential is drawn from a Guassian distribution with zero mean and variance Δ (blue open circles, yellow crosses) or a uniform distribution with the same mean and variance (green filled circles, red crosses). Qualitatively similar results are obtained for other lattice sizes *N*, disorder periods *p*, and gain potential locations *m*_0_ as long as they have a positive threshold. These results are consistent with what we would expect. Introducing disorder suppresses the ${\mathscr{P}}{\mathscr{T}}$-breaking threshold and the threshold distribution *PDF*(*γ*_*PT*_) - a single particle property - is independent of the underlying disorder distribution^41^.

Figure 3(b) shows that these expectations are rather simplistic. For a Gaussian tunneling disorder (blue open circles, yellow crosses), *PDF*(*γ*_*PT*_) is a bell shaped distribution centered about its clean-limit value. It becomes broader when the tunneling disorder strength *λ* is increased, and its center shifts towards the origin. For a uniform disorder (green filled circles, red crosses), we find that *PDF*(*γ*_*PT*_) is now a flat-top distribution approximately centered about its clean-limit value. These results are remarkable because *for a tunneling disorder*, *the threshold distribution PDF*(*γ*_*PT*_) *mimics the disorder distribution and is not universal*.

In one-dimensional Hermitian systems, a random disorder exponentially localizes all states. In transport experiments, this localization is inferred from a scaling analysis of the resistivity in the presence of disorder^29,30^. In optical-waveguide realizations of a Hermitian disordered lattice, it is manifest by a disorder-averaged intensity profile that, after an initial ballistic expansion, develops a steady-state value^24,26,31^. For an initial state on site *k*_0_, the disorder-averaged intensity profile ${I}_{d}(k,t)={|\langle k|\psi (t)\rangle |}_{d}^{2}$ is symmetrically and exponentially localized around that site. Here the subscript *d* denotes averaging over different disorder realizations, |*ψ*(*t*)〉 = *G*(*t*)|*ψ*(0)〉, and the time-evolution operator is *G*(*t*) = exp(−*iHt*) where we have used *ħ* = 1. In the Hermitian case the time-evolution operator is unitary and the total intensity at each time is constant, ${\sum }_{k}\,{I}_{d}(k,t)=1$.

In the ${\mathscr{P}}{\mathscr{T}}$-symmetric disordered case, there are two distinct scenarios. If the gain potential strength is smaller than the minimum threshold value, i.e., *γ* < *γ*_min_ = min_*γ*_{*PDF*(*γ*_*PT*_) > 0}, the system is in the ${\mathscr{P}}{\mathscr{T}}$-symmetric phase for each disorder realization. Therefore, its non-unitary time evolution has bounded intensity oscillations and at long times $Jt\gg 1$, it leads to a quasi steady-state intensity profile *I*_*d*_(*k*) with constant total intensity ${\sum }_{k}\,{I}_{d}(k) > 1$ ^4,33^. When *γ* > *γ*_min_ the system is in the ${\mathscr{P}}{\mathscr{T}}$-broken phase for a fraction of disorder realizations, where the total intensity increases exponentially with time as does the intensity in the neighborhood of the gain site *m*_0_. As a result, the disorder-averaged intensity *I*_*d*_(*k*, *t*) develops a peak at the gain site *m*_0_ whose weight increases with time. We note that in this regime, the intensity *I*_*d*_(*k*, *t*) does not reach a steady state value^4,34,35^.

Figure 3(c–e) encapsulate the effects of periodic disorder on the intensity *I*_*d*_(*k*, *t*). The results are for an *N* = 39 site lattice with on-site disorder, *p* = 10, number of disorder realizations *M* = 10^3^, and an initial state localized at the center of the lattice, |*ψ*(0)〉 = |*k*_0_ = 20〉. Panel (c) shows the disorder-averaged intensity *I*_*d*_(*k*, *t*) for the Hermitian case, *γ* = 0. A periodic disorder leads to a steady-state profile *I*_*d*_(*k*) that is exponentially localized about the initial site *k*_0_ = 20, along with satellite peaks at sites *k* = 20 ± 10 = {10, 30}. These satellite peaks are signatures of extended states that exist in one-dimensional systems with periodic disorder^42,43^. As the disorder strength Δ is increased, the peak intensity of the satellites decreases. We remind the reader that when the disorder is purely random, these satellite peaks are absent.

Panel (d) shows corresponding results for a disordered ${\mathscr{P}}{\mathscr{T}}$-symmetric system with gain potential of strength *γ*/*J* = 0.05 at site *m*_0_ = *p* = 10 (red filled circle); the corresponding loss potential −*iγ* at site ${\bar{m}}_{0}=30$ is also shown (blue filled circle). We see that in addition to the hermitian localization peaks at sites *k* = *k*_0_ mod *p*, a new peak emerges at the gain location. It arises because a disordered system with *γ*/*J* = 0.05 is, sometimes, in the broken ${\mathscr{P}}{\mathscr{T}}$-symmetric phase. Panel (e) shows the disorder-averaged site-intensity profile *I*_*d*_(*k*, *t*) at time *Jt* = 100. In the Hermitian case, the steady-state intensity profile *I*_*d*_(*k*) shows localization peaks at the initial site *k*_0_ = 20 and satellite peaks at sites *k* = {10, 30} (blue filled circles). In the ${\mathscr{P}}{\mathscr{T}}$-symmetric case, the intensity values are essentially unchanged except in the vicinity of the gain site, where the intensity has increased by a factor of five (red open triangles). This interplay between the localization induced by periodic disorder and the broken ${\mathscr{P}}{\mathscr{T}}$-symmetry occurs even if there is no disorder-induced peak at *m*_0_ in the Hermitian limit.

### Disorder induced threshold distribution for gain-loss disorder

Until now, we have confined our attention to Hermitian, periodic, on-site or tunneling disorders that are easily implementable. In this subsection, we will consider the effects of purely imaginary (gain-loss) disorder on the ${\mathscr{P}}{\mathscr{T}}$-symmetric phase. Physically, such a disorder represents random amplifying or absorbing potentials in each waveguide in an otherwise uniform, constant-tunneling waveguide array of size *N*. It is straightforward to see, via perturbative arguments, that if the randomly generated potentials *iγ*_*k*_ are uncorrelated, the ${\mathscr{P}}{\mathscr{T}}$-threshold is zero. If, instead of *N* random potentials {*iγ*_1_, …, *iγ*_*N*_}, one restricts to random, periodic entries {*iγ*_1_, …, *iγ*_*p*_} that are then repeated, the threshold is again zero. Indeed, for a nonzero ${\mathscr{P}}{\mathscr{T}}$-symmetry breaking threshold, the strong, non-local constraint of full ${\mathscr{P}}{\mathscr{T}}$-symmetry is required, i.e., the disorder potential must satisfy $i{\gamma }_{k}=-i{\gamma }_{\bar{k}}$ where index $\bar{k}=N+1-k$ is the reflection counterpart of *k*. We remind the reader that implementing a single pair of balanced gain and loss potentials is experimentally challenging at present, and therefore, implementation of such a disorder, with *N*/2 balanced pairs, is exceedingly difficult in the near future.

The Hamiltonian for a disordered system is given by *H*(*σ*) = *H*_0_ + Γ_*d*_(*σ*) where

8 $${{\rm{\Gamma }}}_{d}(\sigma )=\sum _{k=1}^{[N\mathrm{/2]}}\,i{\gamma }_{k}(|k\rangle \langle k|-|\bar{k}\rangle \langle \bar{k}|).$$

Here [*x*] stands for the integer part of *x* and *iγ*_*k*_ are [*N*/2] i.i.d. random numbers drawn from distribution with zero mean and variance *σ*. We note that the zero mean ensures that the potentials *iγ*_*k*_ in the first half of the lattice approximately average out, i.e. the lattice is locally ${\mathscr{P}}{\mathscr{T}}$-neutral. The other limiting case is *γ*_*k*_ ≥ 0, which corresponds to random *gain*-*only disorder* in the first half of the lattice, with a counterpart loss-only disorder in the second part of the lattice. In this case, we find that ${\mathscr{P}}{\mathscr{T}}$-symmetry breaking threshold is algebraically suppressed to zero^44^. For a locally ${\mathscr{P}}{\mathscr{T}}$-neutral gain-loss disorder, when the variance of the disorder *σ* is small, the spectrum of the disordered Hamiltonian *H*(*σ*) is purely real. It transitions to a complex-conjugate spectrum when the strength exceeds a threshold *σ*_*PT*_. This threshold is disorder-realization dependent, and therefore, we obtain a distribution of the ${\mathscr{P}}{\mathscr{T}}$-symmetry breaking threshold variance.

**Figure 4:**
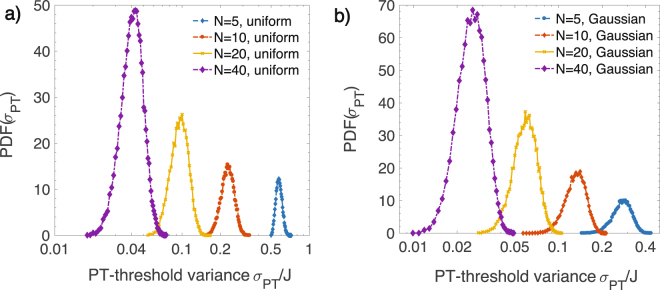
Threshold variance distributions *PDF* (*σ*_*PT*_ for a gain-loss disorder, Eq. (8), as a function of lattice size *N* obtained from *M* = 5 × 10^4^ realizations with (**a**) uniform or (**b**) Gaussian disorder. As the lattice size is doubled, the distribution shifts uniformly along the logarithmic horizontal axis and its width remains unchanged for $N\gg 1$. Note that in a sharp contrast with Fig. 3a,b, these distributions are qualitatively independent of the underlying disorder distribution.

Figure 4 shows such distributions *PDF*(*σ*_*PT*_) for different lattice sizes *N*, obtained with *M* = 5 × 10^4^ realizations drawn from a uniform distribution (a) and Guassian distribution (b). Note that the horizontal axis has a logarithmic scale. We see that the distribution for a Gaussian disorder is wider than that for the uniform disorder. In both cases, as the lattice size *N* is doubled, the threshold probability distribution shifts to smaller values, but its width on the log-scale is essentially unchanged for large lattices, $N\gg 1$. In contrast to the results in Fig. 3(a,b), where the threshold distribution *PDF*(*γ*_*PT*_) depended sensitively on the underlying distribution of Hermitian disorder, these threshold distribution functions *PDF*(*σ*_*PT*_) are qualitatively similar for uniform (a) and Guassian (b) disorders. Thus, the non-Hermitian gain-loss disorder is “traditional”, in the sense that its statistical effects over a large number $M\gg 1$ of disorder realizations are independent of the underlying disorder distribution.

### Beam propagation method analysis

The results for the ${\mathscr{P}}{\mathscr{T}}$-symmetry breaking threshold in disordered lattices in Fig. 1 are based on a tight-binding approximation. In the experimental realizations of such lattices, however, a “site” has a transverse spatial extent, and the tunneling Hamiltonian in Eq. (1) represents a site-discretized version of the spatial second derivative in the continuum Schrödinger (or Maxwell) equation. Therefore, to test that our predictions are not artifacts of the lattice approximation, we obtain the time-evolution of the wave function *ψ*(*x*, *t*) in a waveguide array with realistic parameters^45^ via the beam propagation method (BPM)^46,47^. The continuum Schödinger equation is given by $i{\partial }_{t}\psi =-{\partial }_{x}^{2}\psi /2m+V(x)\psi$. Here, the effective mass is $m={k}_{0}{n}_{0}^{2}/c$, the potential is given by $V(x)=c{k}_{0}[1-n{(x)}^{2}/{n}_{0}^{2}]$, *n*_0_ is the cladding index of refraction, *c* is the speed of light in vacuum, *n*(*x*) is the position-dependent index of refraction in the waveguide array, and *k*_0_ = 2*π*/*λ* is the wave number of the rapidly varying part of the electric field $E(x,z,t)=\exp \,[i{k}_{0}z-(c{k}_{0}/{n}_{0})t]\,\psi (x,t)$ which satisfies the Maxwell equation.

**Figure 5:**
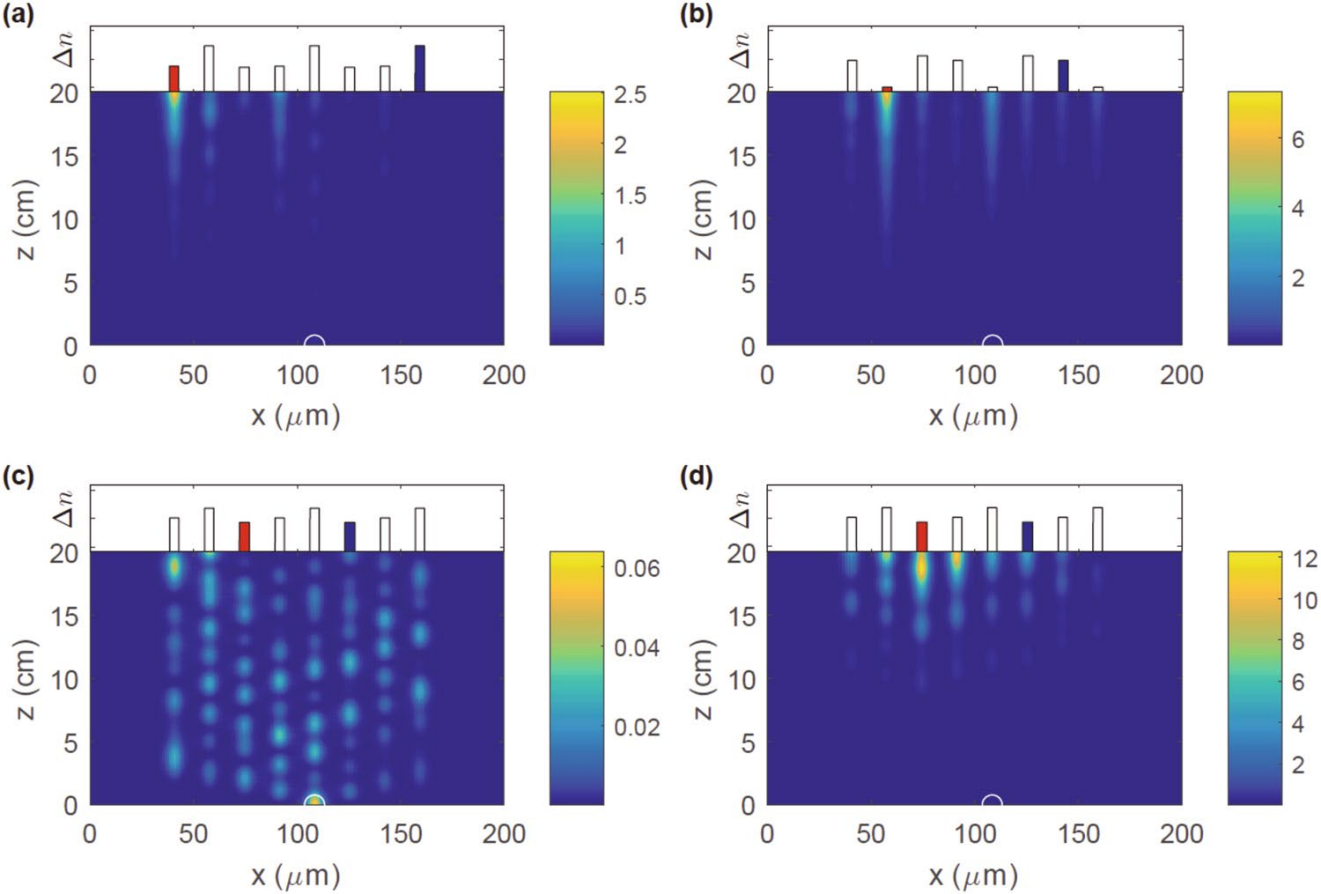
BPM simulations of wave packet propagation in an *N* = 8 waveguide lattice in the presence of on-site disorder with period *p* = 3. The system parameters are *λ* = 633 nm, cladding index *n*_0_ = 1.45, waveguide width *W* = 10 *μ*m, and uniform waveguide separation *d* = 16.9586 *μ*m. The bar-chart at the top of each panel shows a random, periodic index-contrast distribution Δ*n*(*x*). The vertical scale in each bar-chart ranges from 4.8 × 10^−4^ to 5.2 × 10^−4^. (**a**) For a gain potential with strength *γ* = 0.7 cm^−1^ on the first site, the intensity *I*(*x*, *z*) shows a ${\mathscr{P}}{\mathscr{T}}$-symmetry broken state. (**b**) With the same gain potential on the second site, the system is again in the ${\mathscr{P}}{\mathscr{T}}$-broken phase. (**c**) With the same gain-potential on site *m*_0_ = 3 = *p*, the system is in the ${\mathscr{P}}{\mathscr{T}}$-symmetric phase, as is also shown by site intensities that are one to two orders of magnitude smaller. (**d**) When the gain potential at site *m*_0_ = 3 is doubled to *γ* = 1.4 cm^−1^, the system is in the ${\mathscr{P}}{\mathscr{T}}$-symmetry broken state. Note that the index-contrast profiles in (**c**,**d**) are the same. The BPM analysis confirms the predictions for zero and positive ${\mathscr{P}}{\mathscr{T}}$-thresholds in the presence of random, periodic disorder.

The index of refraction *n*(*x*) differs from that of the cladding only within each waveguide. In the limit of small contrast, *n*(*x*) = *n*_0_ + Δ*n* with ${\rm{\Delta }}n/{n}_{0}\sim {10}^{-4}\ll 1$, the potential term becomes linearly proportional to the index contrast, i.e., *V*(*x*) = 2*ck*_0_Δ*n*/*n*_0_, and we implement the gain and loss potentials by adding appropriate imaginary parts to the index contrast. Figure 5 shows representative results of such simulations for an *N* = 8 waveguide-lattice in the presence of an on-site disorder with period *p* = 3. The initial state, marked by a white semicircle, is a normalized Gaussian with width *σ* = *W*/2 in the 5th waveguide, where *W* is the width of each waveguide. Each panel shows the time- and space-dependent intensity *I*(*x*, *z* = *ct*/*n*_0_) where we have switched to the distance along the waveguide *z* = *ct*/*n*_0_ as a measure of time for an easier comparison with experiments. The bar-chart at the top of each panel shows a randomly generated index contrast Δ*n*(*x*) with period *p* = 3. The gain-potential waveguide is shown by a red bar, the reflection-symmetric lossy waveguide is shown by a blue bar, and the linear scale on the vertical axis in each bar-chart ranges from Δ*n* = 4.8 × 10^−4^ to Δ*n* = 5.2 × 10^−4^.

The intensity plot *I*(*x*, *z*) in Fig. 5(a) is for a gain potential *γ* = 0.7 cm^−1^ in the first waveguide, *m*_0_ = 1. It shows that at long times, *z* ≥ 10 cm, the intensity is largely confined to the gain waveguide and the system is in the broken ${\mathscr{P}}{\mathscr{T}}$-symmetry phase. Panel (b) has the intensity plot with the same gain in the second waveguide, *m*_0_ = 2; it also shows intensity localized in the gain waveguide and thus indicates that the system is in the ${\mathscr{P}}{\mathscr{T}}$-broken phase. In each case, we note that the maximum intensity *I*(*x*, *z*) is larger than the average intensity $I\sim 1/N=0.125$ expected in each waveguide in the Hermitian limit. Panel (c) shows *I*(*x*, *z*) with the same gain in the third waveguide, *m*_0_ = 3. It is clear from the intensity plot that the system is in the ${\mathscr{P}}{\mathscr{T}}$-symmetric phase. Panel (d) shows that when the gain potential is doubled, i.e. *γ* = 1.4 cm^−1^, the system enters a ${\mathscr{P}}{\mathscr{T}}$-broken phase and the resultant intensity is localized largely to the gain waveguide. The results presented in Fig. 5 are generic and demonstrate that our findings of zero or positive ${\mathscr{P}}{\mathscr{T}}$-thresholds in disordered lattices are robust (Fig. 1). We emphasize that it is very difficult to determine the actual value of a positive ${\mathscr{P}}{\mathscr{T}}$-breaking threshold from the BPM analysis; the closer one is to the threshold - from below or from above - the longer is the time evolution required to distinguish between bounded oscillatory behavior and exponentially increasing behavior.

## Discussion

In this paper we have introduced non-Hermitian lattice models with balanced gain and loss that are robust against random, periodic disorder. We have uncovered a veiled symmetry that is exhibited by eigenfunctions of such disordered, Hermitian lattices. This symmetry is phase-sensitive, and it ensures equal weights at specific reflections-symmetric sites, but not equal wave functions^40^. Therefore, any phase-insensitive observable will reflect the signatures of this symmetry. Experimentally, the models studied here can be realized in coupled waveguide arrays with one gain waveguide and one lossy waveguide. Ideally, if the on-site potentials or tunneling amplitudes are tunable - for example, via voltage-controlled top-gate heaters - it will permit experimental investigations of interplay between localization due to a periodic disorder and the ${\mathscr{P}}{\mathscr{T}}$-symmetry breaking transition.

Mathematically, the lattice models considered here correspond to tridiagonal matrices with Hermitian, random, periodic entries, in addition to non-Hermitian, fixed, gain-loss potential entries along the main diagonal. The statistical properties of eigenvalues of such matrices are essentially unexplored. In particular, the dependence of the threshold distribution *PDF*(*γ*_*PT*_) on the source and the distribution of disorder is, at this point, poorly understood. A generalization of these models to non-sparse matrices with a positive ${\mathscr{P}}{\mathscr{T}}$-symmetry breaking threshold^48^, will provide an approach to investigate the spectral properties random, ${\mathscr{P}}{\mathscr{T}}$-symmetric matrices with real spectra.
